# Pathological complete response (pCR) with or without the residual intraductal carcinoma component following preoperative treatment for pancreatic cancer: Revisiting the definition of “pCR” from the prognostic standpoint

**DOI:** 10.1002/ags3.12288

**Published:** 2019-09-20

**Authors:** Daisaku Yamada, Hidenori Takahashi, Kei Asukai, Shinichiro Hasegawa, Akira Tomokuni, Hiroshi Wada, Hirofumi Akita, Masayohi Yasui, Hiroshi Miyata, Osamu Ishikawa

**Affiliations:** ^1^ Department of Gastroenterological surgery Osaka International Cancer Institute Osaka Japan

**Keywords:** intraductal carcinoma component, pancreatic ductal adenocarcinoma, pathological complete response

## Abstract

**Background and Aim:**

There are no previous reports describing the prognostic significance of the residual intraductal carcinoma component (carcinoma in situ [CIS]) following preoperative treatment for pancreatic ductal adenocarcinoma (PDAC). The aim of the present study was to investigate the prognostic significance of a minimal residual CIS in cases with complete absence of an invasive component after preoperative treatment for PDAC.

**Methods:**

Eighty‐one of 594 PDAC patients with preoperative treatment and subsequent surgery in our institute showed remarkable remission in the invasive component, which included 48 patients with the minimal residual invasive component (Min‐inv group) and 33 with absence of an invasive component (No‐inv group). We assessed the survival of these patients in association with the presence or absence of an invasive component and intraductal CIS.

**Results:**

Five‐year overall survival in the No‐inv group patients was significantly better than that of the Min‐inv group patients (82%/66%, *P* = .041). Among the 33 patients in the No‐inv group, residual CIS was observed in 16 patients (CIS‐positive group), and the remaining 17 patients had no residual CIS (CIS‐negative group). There was no significant difference in survival between patients in the CIS‐positive and CIS‐negative groups (92%/78%, *P* = .31).

**Conclusions:**

Residual CIS in the absence of an invasive component after preoperative treatment does not yield a prognostic impact after receiving perioperative treatment for PDAC. It might be reasonable to define pathological complete response (pCR) from the prognostic standpoint as follows: pCR is the complete absence of an invasive carcinoma component regardless of residual CIS.

## Abstract 2

We investigated the survival outcome of pancreatic ductal adenocarcinoma patients showing complete absence of an invasive component in relation to those with minimal residual carcinoma in situ (CIS) after preoperative therapy; complete regression of the invasive component, regardless of the presence or absence of residual CIS, was a significant indicator for preferable prognosis.

## Graphical abstract

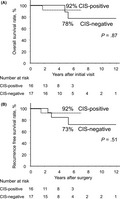

## 1 INTRODUCTION

Pancreatic ductal adenocarcinoma (PDAC) is a lethal disease because tumor cells have a tendency to spread to the surroundings and/or distant organs and become systematic disease from an early stage.^1^, ^2^, ^3^ A solely surgical approach for PDAC is able to potentially cure this disease in few patients.^4^, ^5^ The surgery‐alone strategy provides the minimum survival benefit in the majority of patients with localized PDAC (ie, resectable [R] or borderline resectable [BR] stage);^6^ thus, multimodal strategies, including surgery plus pre‐/postoperative therapies, have been attempted to improve surgical outcomes in patients with R/BR‐PDAC.^7^, ^8^, ^9^, ^10^, ^11^, ^12^, ^13^, ^14^, ^15^, ^16^, ^17^ In our institute, we conducted a multimodal treatment strategy consisting of surgical resection following preoperative treatment and subsequent postoperative treatment for patients with PDAC.^7^, ^8^ Preoperative therapies have certain possible clinical benefits,^18^, ^19^, ^20^, ^21^, ^22^ including a locoregional effect, early administration of systemic therapy and possible reduction in systemic recurrence after surgery. Histopathological evaluations of resected specimens after preoperative treatment (ie, histopathological response) are one of the indicators to assess the efficacy of preoperative treatment, and the histopathological response has been investigated in association with survival after surgery. Several reports have shown a remarkable response to preoperative therapy for PDAC, indicating a significantly preferable patient prognosis.^11^, ^12^, ^13^, ^14^, ^15^, ^16^

Pathological complete response (pCR) is recognized as the ultimate form of histopathological response, and previous reports showed that 1.6%‐13% of PDAC patients showed pCR to preoperative therapy (the total number of patients with pCR was 44 cases in those studies),^15^, ^16^, ^21^, ^22^, ^23^ pCR is defined as the observation of complete absence of an invasive cancer component in the post‐treatment tissue.^24^, ^25^ However, how to interpret the minimal residual intraductal component (CIS, carcinoma in situ) is controversial in relation to the definition of pCR. The recently published Japanese classification first referred to residual CIS after preoperative treatment for PDAC^26^ and, in previous reports, patients showing complete remission of the invasive component with minimal residual CIS were usually regarded as having pCR as also shown in the new Japanese classification.^15^, ^16^, ^21^ Nevertheless, there are no reports clearly showing the significance of residual CIS. In breast cancer, previous investigations for the prognostic significance of residual cancer cells in the intraductal component after preoperative therapy showed controversial results, and interpretation of minimal residual CIS remains undetermined in the definition of pCR.^27^, ^28^, ^29^, ^30^

In this context, no report has addressed whether pCR applies to cases of minimal residual CIS in the preoperative treatment strategy for PDAC. The clinical significance of minimal residual CIS remains unclear from the prognostic standpoint, whereas a few reports have indicated that the prognosis of patients with the minimal residual invasive component was significantly worse than that of patients showing complete absence of an invasive component in post‐treatment tissues after preoperative treatment for PDAC.^15^, ^16^, ^21^ Therefore, we conducted the present study with the aim of investigating the clinical significance of minimal residual CIS in patients with the absence of an invasive component with reference to the prognostic impact of the minimal residual invasive component in a resected specimen after preoperative chemoradiation therapy for R/BR‐PDAC. From the prognostic viewpoint, we evaluated whether it is appropriate that patients with residual PDAC cells in the intraductal component only are classified as having pCR status.

## 2 METHODS

### 2.1 Patients

We retrospectively investigated all patients with histologically confirmed PDAC who received an R0 resection following preoperative therapy in our institute from January 2003 to December 2016, and 594 patients were included in this study. Our main strategy for PDAC was not changed during this 14‐year period.^7^, ^8^ Patients with PDAC growing outside the pancreas but not into nearby major blood vessels (T3 stage of UICC 7th edition including the R/BR stage of NCCN classification ver. 1, 2019) are usually recommended to undergo preoperative chemoradiation therapy (CRT, chemotherapy and radiation as conventional external‐beam radiotherapy) for 2‐3 months before surgery.

### 2.2 Preoperative therapy

All patients included in the present study received preoperative therapy. During the observation period, several preoperative therapies were used according to the relevant clinical studies. CRT was mainly used, and chemotherapy regimens were selected at our discretion, mainly depending on the national availability of chemo‐drugs. In the first stage, patients were treated by single gemcitabine agent plus radiation.^7^ After that, gemcitabine‐based combination regimens (gemcitabine/nab‐paclitaxel or gemcitabine/S‐1) concomitant with radiation therapy were often used.^8^, ^10^ Patients unsuitable for gemcitabine therapy or for radiation therapy were treated with single S‐1 agent plus radiation or with gemcitabine‐based combination chemotherapy omitting radiation therapy. All of these patients provided written informed consent for participation.

### 2.3 Surgery

Computed tomography (CT) or magnetic resonance imaging (MRI) was carried out to judge the resectability of PDAC within the 4 weeks before surgery. Patients who proceeded to surgery underwent pancreaticoduodenectomy, distal pancreatectomy, or total pancreatectomy as determined by the location and extent of the tumor. Vascular resections were done if involvement of the superior mesenteric vein, portal vein, celiac axis, or hepatic artery was substantial. Vascular reconstruction was preferentially carried out by primary repair. Patch venous repair or interposition grafting was used only when primary repair was not feasible.

### 2.4 Pathological examination

**Figure 1:**
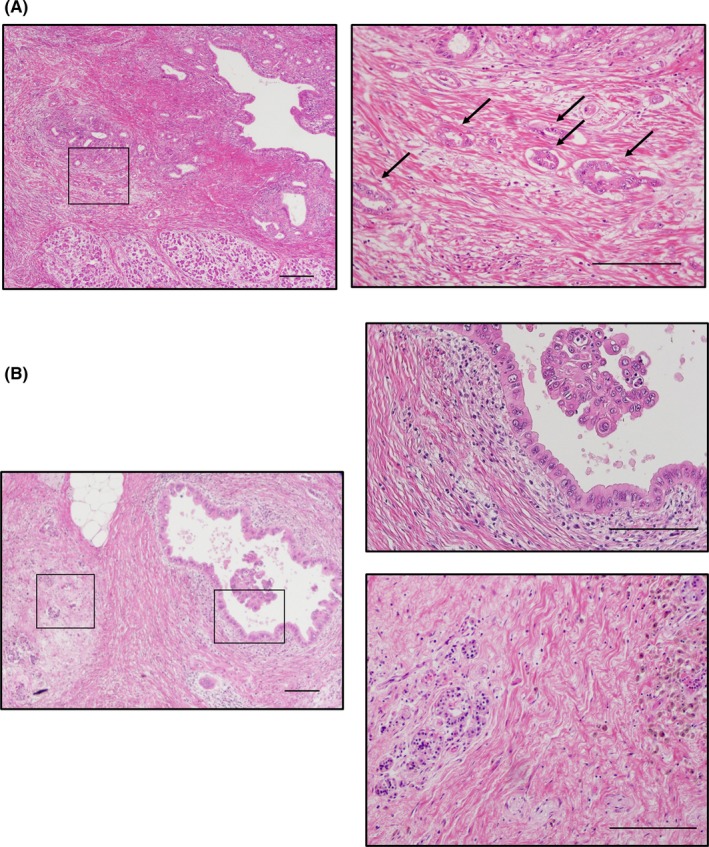
Histological findings showed only a small number of viable cells in the resected tissue, and the regression grade was estimated to be <10% in the invasive component (Min‐inv), or no viable cancer cells were found in the invasive component (No‐inv). Patients in the “No‐inv” group were further classified into two groups based on the presence or absence of residual cancer cells in the intraductal component, including high‐grade dysplasia or cancer cells in situ (CIS‐negative group/CIS‐positive group). Panels show representative histological findings of Min‐inv (A) and CIS‐positive (B). A, Left panel shows the main pancreatic duct and surrounding pancreatic tissue of Min‐inv. Scale bar, 200 μm. Right panel is the high‐power field indicated in the left panel by a rectangle. Arrows indicate residual cancer cells in the invasive cancer component. Scale bar, 200 μm. B, Left panel shows the main pancreatic duct and surrounding pancreatic tissue of the No‐inv group with cancer cells in situ. Rectangles indicate the locations of adjacent panels of high‐power fields. Scale bar, 200 μm. Right upper panel is the high‐power field of the main pancreatic duct that is indicated in the left panel, and cancer cells in situ are shown. Scale bar, 200 μm. Right lower panel is the high‐power field of the invasive cancer component that is indicated in the left panel, and there were no cancer cells. Scale bar, 200 μm

Pathological examinations were carried out as previously described.^2^ Human PDAC samples were obtained with institutional review board approval and fixed in 10% formalin for 48 hours, embedded in paraffin, the specimen cut into 3‐4 mm width and sectioned into 3.5‐μm slices. Formalin‐fixed, paraffin‐embedded tissue sections that included PDAC were deparaffinized, hydrated, and stained with hematoxylin and eosin. These samples were adequately evaluated and diagnosed by expert pathologists in our institute, and the effect of preoperative therapy was estimated. In this study, we divided the patients into groups based on two components: the invasive component and the intraductal component. When focusing on the invasive component, patients were divided according to the presence of residual cancer cells in the invasive component. Patients were assigned to the “No‐inv” group when we did not detect any viable cancer cells in the invasive component regardless of the presence of residual cancer cells in the pancreatic duct (these patients corresponded to Grade 0 in the College of American Pathologists [CAP] system,^25^ Grade IV in the Evans classification^24^). Patients were assigned to the “Min‐inv” group when there were only a small number of viable cells in the resected tissue, and the regression grade was estimated at less than 10% in the invasive component (these patients corresponded to Grade 1 in CAP, Grade III in the Evans classification). Focusing on minimal residual cancer cells in the intraductal component only, “No‐inv” group patients were further classified into two groups based on the presence or absence of residual cancer cells in the intraductal component, including high‐grade dysplasia or cancer cells in situ (CIS‐negative group/CIS‐positive group; Figure 1).

### 2.5 Adjuvant chemotherapy and follow up

We carried out liver perfusion chemotherapy (LPC) through the portal vein followed by surgery, as reported previously.^7^ All patients received LPC from the first day after surgery if their condition allowed it, and the perfusion was continued for 4 weeks in the hospital. After discharge from the hospital, patients were highly encouraged to undergo systemic adjuvant chemotherapy. From 2003 to 2011, patients were mainly treated with gemcitabine, based on the results of the CONKO‐001 study.^4^, ^31^ Subsequently, S‐1 was used as an adjuvant agent, according to the results of the JASPAC‐001 study.^5^

Follow‐up observations were carried out as described previously.^8^ To investigate recurrence, three types of examination were carried out every 3‐4 months: a routine physical examination; laboratory tests, including the analysis of the serum level of CA19‐9 (tumor marker); and radiological imaging, including chest and abdominal CT (or MRI). Date of recurrence was defined as the date on which the investigator detected recurrence on an image or in a biopsy specimen. The last follow‐up date was June 2018, and the median observation time after initial diagnosis of patients was 39.1 months.

### 2.6 Evaluation of clinicopathological features

We investigated various preoperative variables, including patient characteristics, tumor factors and treatment factors (Table 1). We collected common patient parameters and all tumor information, including initial findings at the first visit and before surgery (after preoperative therapy), and the pathological diagnoses were estimated using the resected specimens.

**Table 1:** Clinicopathological characteristics in 81 patients showing remarkable regression by preoperative chemoradiation therapy

	Median ± SD or n
Patient characteristics	
Age (y)	64.0 ± 8.9
Gender (M/F)	44/37
Tumor factors	
Location (Ph/Pb or Pt)	46/35
Initial resectability (R/BR)	39/42
Initial tumor diameter (mm)	22.3 ± 9.8
Tumor diameter before surgery (mm)	15.2 ± 9.7
CA19‐9 (U/mL): Initial visit	676.4 ± 3211.6
CA19‐9 (U/mL): Before surgery	80.3 ± 236.4
ypN, UICC ver. 8th (0/1/2)	77/2/2
Differentiation (well/moderate/poor)	13/30/5
Histological invasion in micro tissue	
Micro vascular (−/+)	79/2
Nerve (−/+)	72/9
Presence of remnant invasive component (Min‐inv/No‐inv)	**48/33**
Presence of remnant intraductal component (CIS‐negative/CIS‐positive)	**56/25**
Treatment factors	
CRT protocol (GRT/G‐based + RT/S‐1RT)	54/22/5
Surgery type (PD/DP/TP)	49/29/3
Adjuvant chemotherapy	
LPC (−/+)	26/55
Systemic chemotherapy (−/+)	39/42

Abbreviations: BR, borderline resectable; CIS, carcinoma in situ; CRT, chemoradiotherapy; DP, distal pancreatectomy; G‐based + RT, gemcitabine‐based combination regimens (gemcitabine/nab‐paclitaxel or gemcitabine/S‐1) concomitant with radiation therapy; GRT, single gemcitabine agent plus radiation treatment; LPC, liver perfusion chemotherapy; Pb, pancreas body; PD, pancreaticoduodenectomy; Ph, pancreas head; Pt, pancreas tail; R, resectable; S‐1RT, single S‐1 agent plus radiation treatment; TP, total pancreatectomy; UICC, Union for International Cancer Control.

The topic of this manuscript, 'No‐inv/Min‐inv' and 'CIS‐negative group/CIS‐positive' groups, were highlighted with bold characters.

### 2.7 Statistical analysis

Data are expressed as mean ± standard deviation. The chi‐squared test and Fisher's exact test were used for comparing categorical variables, as appropriate. A Kaplan‐Meier analysis and log‐rank test were used to construct survival curves and to evaluate differences in univariate analysis. Logistic regression was carried out for both the multivariate analysis and the partition analysis of the detected factors. All analyses were done using the JMP 13 software program (SAS Institute. *P* values <.05).^32^

### 2.8 Ethical considerations

This study was carried out at Osaka International Cancer Institute, Japan, and was approved by the ethics committee of our institution (no. 18195).

## 3 RESULTS

### 3.1 Tumor regression with preoperative chemoradiation therapies

Preoperative chemoradiation therapies showed remarkable tumor regression in a number of the patients retrospectively investigated from 2003 to 2016. Among the 594 patients with PDAC who received R0 resection following preoperative therapy, 81 patients (13.6%) showed remarkable regression, categorized into the Min‐inv group (48 patients, 8.1%) or the No‐inv group (33 patients, 5.6%). In these 81 patients, average age at time of surgery was 64 years, and 54.3% were male. Median number of days of preoperative therapy treatment was 122, and all of the patients received radiation therapy. Average diameter of initial tumor was 22.3 mm; 42 patients (51.9%) were initially categorized as having BR‐PDAC based on the initial image, and 13 patients underwent concomitant vascular resection with surgery (Table 1). Sixteen patients had CIS, and 48.5% of the patients in the No‐inv group were categorized into the CIS‐positive group.

### 3.2 Absence of tumor cells in the invasive component after preoperative therapy indicated significantly better prognosis

**Figure 2:**
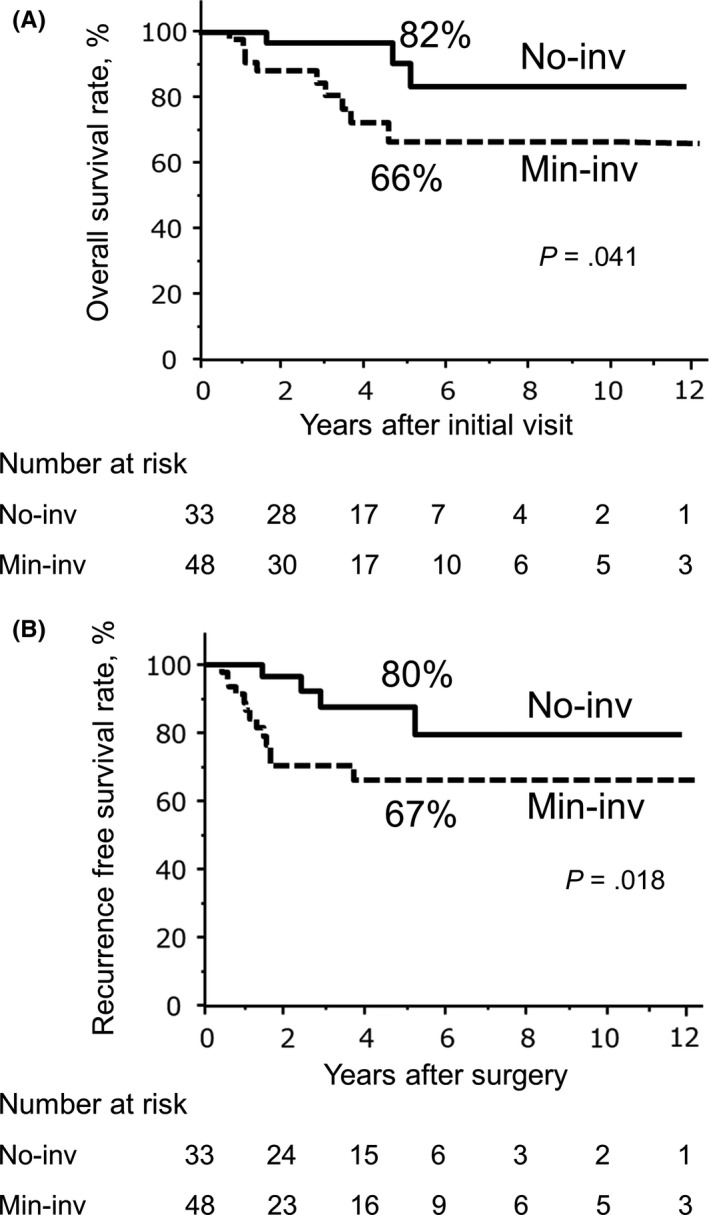
Kaplan‐Meier survival curves of overall survival (OS) and recurrence‐free survival (RFS) of patients showing remarkable remission with preoperative therapy. OS after first visit (A) and RFS after surgery (B) curves of 81 patients with pancreatic ductal adenocarcinoma are shown. Groups are divided according to histological findings of the Min‐inv group (n = 48) and No‐inv group (n = 33). Five‐year OS: No‐inv and Min‐inv: 82% and 66%, respectively, *P* = .041. Five‐year RFS: No‐inv and Min‐inv: 80% and 67%, respectively, *P* = .018

To elucidate the clinical impact of a small number of residual cancer cells in the invasive component, we compared the outcomes of the patients in the Min‐inv group and in the No‐inv group. Median observation time was 39.1 months (3.8‐173.2 months). Overall survival (OS) of patients in the Min‐inv group was significantly shorter than that of patients in the No‐inv group (Figure 2A, OS at 5 years of No‐inv and Min‐inv groups were 82% and 66%, respectively, *P* = .041), and recurrence‐free survival (RFS) was also significantly longer in the No‐inv group than in the Min‐inv group (Figure 2B, 5‐year RFS in the No‐inv and Min‐inv groups: 80% and 67%, respectively, *P* = .018.). Twenty‐nine (87.9%) patients in the No‐inv group were free from PDAC recurrence after surgery, whereas approximately 12% (four patients) developed recurrence. All of these four patients developed lung metastasis at over 1 year after surgery, two of them simultaneously developed bone metastasis, but no patients developed local recurrence or liver metastasis. One of the four patients in the No‐inv group who developed PDAC recurrence showed residual CIS in the resected specimen, and three of them did not receive systemic adjuvant chemotherapy after surgery regardless of the presence of remnant CIS.

There was no significant difference in the comparison of clinicopathological parameters of the two cohorts, except for age and rate of administration of adjuvant systemic chemotherapy (Table 2). Average patient age was significantly lower and the rate of giving systemic chemo‐drugs as adjuvant therapy was significantly higher in the Min‐inv group than in the No‐inv group. Tumor size, resectability and value of the tumor marker did not differ between the cohorts at the initial visit. There was no difference in OS between patients with initial R‐PDAC and patients with initial BR‐PDAC (5‐year survival rate of R‐PDAC 73%, BR‐PDAC 81%, *P* value .96) in this study that investigated patients who showed remarkable regression with preoperative therapies. Type of preoperative therapy did not tend to change the rate of absence of cancer cells in the invasive component, and the value of the tumor marker after preoperative therapy did not distinguish patients in the No‐inv group from patients in the Min‐inv group. Postoperative adjuvant treatments did not show obvious impact on the OS in this cohort: the 5‐year survival rates of patients who received either postoperative LPC or systemic adjuvant chemotherapy, or both, and those who did not receive any adjuvant treatment were 73% and 84%, respectively (*P* = .98). More specifically, the 5‐year survival rates of the patients who received postoperative LPC and those who did not were 76% and 79%, respectively (*P* = .36), and those of patients who received systemic adjuvant chemotherapy and those who did not were 68% and 84%, respectively (*P* = .14).

**Table 2:** Comparison of clinicopathological parameters among the cohorts of Min‐inv patients and No‐inv patients

	Univariate analysis		
	Min‐inv	No‐inv	*P* value
	Median ± SD or n		
Patient characteristics			
Age (y)	**61.9 ± 1.2**	**67.0 ± 1.5**	**<.01**
Gender (M/F)	28/20	16/17	.38
Tumor factors			
Location (Ph/Pb or Pt)	27/21	19/14	.91
Initial resectability (R/BR)	23/25	16/17	.59
Initial tumor diameter (mm)	23.7 ± 1.4	20.3 ± 1.7	.14
CA19‐9 (U/mL): Initial visit	982.3 ± 463.4	231.5 ± 558.8	.30
CA19‐9 (U/mL): Before surgery	52.8 ± 34.0	120.4 ± 41.0	.21
ypN, UICC ver. 8 (0/1/2)	44/2/2	33/0/0	.72
Presence of remnant intraductal component (CIS‐negative/CIS‐positive)	39/9	17/16	.15
Treatment factors			
CRT protocol (GRT/G‐based + RT/S‐1RT)	31/14/3	23/8/2	.94
Surgery type (PD/DP/TP)	29/17/2	20/12/1	.96
Adjuvant chemotherapy			
LPC (−/+)	13/35	13/20	.25
Systemic chemotherapy (−/+)	**18/30**	**21/12**	**.02**

Abbreviations: BR, borderline resectable; CIS, carcinoma in situ; CRT, chemoradiotherapy; DP, distal pancreatectomy; G‐based + RT, gemcitabine‐based combination regimens (gemcitabine/nab‐paclitaxel or gemcitabine/S‐1) concomitant with radiation therapy; GRT, single gemcitabine agent plus radiation treatment; LPC, liver perfusion chemotherapy; Pb, pancreas body; PD, pancreaticoduodenectomy; Ph, pancreas head; Pt, pancreas tail; R, resectable; S‐1RT, single S‐1 agent plus radiation treatment; TP, total pancreatectomy; UICC, Union for International Cancer Control.

The number of factors which showed significant difference were highlighted with bold characters.

### 3.3 Presence of CIS did not affect the prognosis of patients with no viable cells in the invasive component

**Figure 3:**
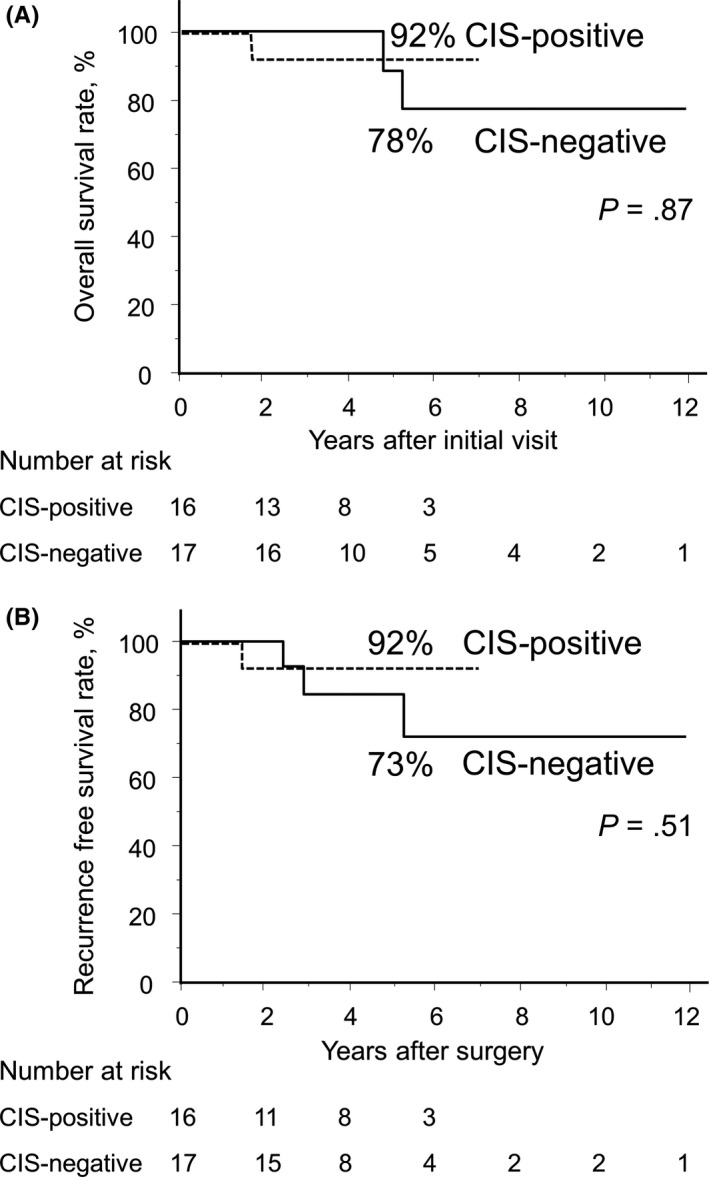
Kaplan‐Meier survival curves of overall survival (OS) and recurrence‐free survival (RFS) of patients with pathological complete response (pCR). OS after first visit (A) and RFS after surgery (B) curves of 33 patients with pancreatic ductal adenocarcinoma are shown. Groups are divided according to histological findings of the presence/absence of residual cancer cells in the intraductal component, including high‐grade dysplasia or cancer cells in situ (CIS‐positive, n = 16/CIS‐negative, n = 17). Five‐year OS: CIS‐positive and CIS‐negative: 92% and 78%, respectively, *P* = .87. Five‐year RFS: CIS‐positive and CIS‐negative: 92% and 73%, respectively, *P* = .51

To investigate the role of residual cancer cells in the intraductal component only, we compared the prognosis of CIS‐positive and CIS‐negative patients in the No‐inv group. Median observation time was 47.8 months (5.3‐138.9 months), and there was no difference in either OS or RFS between these groups (Figure 3, CIS*‐*positive and CIS*‐*negative groups: 5‐year OS: 92% and 78%, respectively, *P* = .87; 5‐year RFS: 92% and 73%, respectively, *P* = .51). Comparison of the clinicopathological parameters did not detect any significant difference between these groups (Table 3).

**Table 3:** Comparison of clinicopathological parameters among the cohorts of CIS‐negative patients and CIS‐positive patients

	Univariate analysis		
	CIS‐negative	CIS‐positive	*P* value
	Median ± SD or n		
Patient characteristics			
Age (y)	65.0 ± 1.9	69.3 ± 1.9	.12
Gender (M/F)	11/6	5/11	.06
Tumor factors			
Location (Ph/Pb or Pt)	9/8	10/6	.58
Initial resectability (R/BR)	7/10	9/7	.39
Initial tumor diameter (mm)	22.9 ± 2.4	17.6 ± 2.4	.12
CA19‐9 (U/mL): Initial visit	233.8 ± 102.0	228.9 ± 104.9	.98
CA19‐9 (U/mL): Before surgery	160.4 ± 83.2	77.9 ± 85.8	.50
Treatment factors			
CRT protocol (GRT/G‐based + RT/S‐1RT)	12/4/1	11/4/1	.99
Surgery type (PD/DP/TP)	10/7/0	10/5/1	.96
Adjuvant chemotherapy			
LPC (−/+)	8/9	5/11	.25
Systemic chemotherapy (−/+)	11/6	10/6	.90

Abbreviations: BR, borderline resectable; CIS, carcinoma in situ; CRT, chemoradiotherapy; DP, distal pancreatectomy; G‐based + RT, gemcitabine‐based combination regimens (gemcitabine/nab‐paclitaxel or gemcitabine/S‐1) concomitant with radiation therapy; GRT, single gemcitabine agent plus radiation treatment; LPC, liver perfusion chemotherapy; Pb, pancreas body; PD, pancreaticoduodenectomy; Ph, pancreas head; Pt, pancreas tail; R, resectable; S‐1RT, single S‐1 agent plus radiation treatment; TP, total pancreatectomy; UICC, Union for International Cancer Control.

## 4 DISCUSSION

Pancreatic ductal adenocarcinoma is generally considered a chemo‐ and radio‐resistant cancer, and pCR has rarely been observed in practice. Evans et al^23^ reported the outcomes of gemcitabine‐based CRT for resectable PDAC in which pCR was observed in only 1.6% (1/64). Recent advances in chemotherapy and radiation therapy for PDAC provided more favorable preoperative treatment effects; thus, the frequency of pCR might increase as a result of preoperative treatment strategies for PDAC. Barenboim et al^22^ reported that the frequency of pCR was 13% in patients who received FOLFIRINOX preoperatively. He et al^16^ described a high rate of pCR in patients who were treated mainly by a multiagent preoperative treatment protocol, including FOLFIRINOX, in which the frequency of pCR was 10% (19/186). Although the incidence of cases with remarkable histopathological response (eg, pCR) is increasing, there has been uncertainty in the definition of pCR, namely, in reference to the presence of residual minimal CIS. The histopathological response scheme usually uses a four‐tiered grading system focusing on the invasive component, such as the grading scheme by Evans et al and the CAP system.^24^, ^25^ However, these grading systems do not include clear descriptions of how to interpret residual CIS. The Japan Pancreas Society (JPS) recently published a new classification for the histopathological response (4th edition), and they proposed that cases where only the intraductal components remain in the post‐treatment tissue are classified as pCR.^26^ However, the JPS system also mentioned that the significance of the residual CIS remains unclear and requires further investigation.

The present study indicated that the presence of minimal residual CIS did not have a significant prognostic impact in patients with absence of an invasive component after preoperative CRT, whereas a minimal residual invasive component was significantly associated with impaired survival. We may draw two interpretations from these results. First, it would be reasonable to categorize cases with the absence of an invasive component after preoperative treatment for PDAC as “pCR” regardless of the presence or absence of residual CIS from the prognostic standpoint. Our results endorse the concept of pCR used in the previous reports and the proposal of the JPS definition of pCR, presenting detailed clinical features of pCR in relation to the presence or absence of the residual CIS.^15^, ^16^, ^21^, ^26^ Second, we should be aware of the prognostic significance of the residual invasive component, even if it is minimal. He et al^16^ showed similar results to ours, in which 29 patients with minimal residual invasive tumors (“nCR” in their report) after CRT for locally advanced PDAC showed worse prognosis than 19 patients with absence of an invasive component (“pCR”) and an equivalent prognosis to the limited responders. A majority of previous reports analyzed the outcomes of preoperative treatment, with both pCR cases and the cases with minimal residual invasive component being combined into a single category, possibly due to the small number of pCR cases.^11^, ^12^, ^13^, ^14^ However, as the number of pCR cases increases in the modern era of potent preoperative treatment for PDAC, pCR cases and cases with minimal residual invasive component should be analyzed separately from each other to more appropriately evaluate the efficacy of preoperative treatment for PDAC because of the distinct difference in prognostic impact between complete absence and the minimal residual invasive component.

Considerations of the mechanisms of the presence or absence of residual CIS in pCR cases are of interest. Some authors reported that approximately half of PDAC cases were accompanied by a rich intraductal component, whereas the remaining half of PDAC cases were not.^33^, ^34^, ^35^ In this context, the pCR cases with absence of CIS might originate from tumors that were not originally accompanied by CIS before preoperative treatment, showing complete response in the invasive component to preoperative treatment. Certainly, there is a possible scenario regarding pCR cases with absence of CIS: the invasive PDAC accompanying CIS before preoperative treatment might show a complete response in both the invasive and intraductal components to preoperative treatment. In breast cancer, the characteristics and number of cancer cells in the intraductal component are changed by preoperative therapy, and tumor cells in the intraductal component show more resistance to preoperative treatment.^27^, ^28^ Based on this concept, it can be postulated that PDAC with CIS before preoperative treatment shows a complete response in the invasive component and fails to achieve a complete response in the intraductal component as a result of the more resistant nature of the intraductal component to preoperative treatment, resulting in the observation of pCR with residual CIS. Further investigations are required to address the problems of pathogenesis of residual CIS in pCR cases. It would be interesting to compare the molecular profiles of residual CIS and the invasive components obtained before preoperative treatment by endoscopic ultrasonography‐fine‐needle aspiration (EUS‐FNA) and other methods in cases with or without residual CIS to gain a deeper understanding of the underlying mechanisms associated with the chemo‐ or radio‐resistance of PDAC.

Recurrence in pCR cases is an important problem to be addressed. In the current study, four of 33 pCR cases experienced tumor recurrence after surgery following preoperative treatment, and lung recurrences were observed in all of those cases. Several similar cases of PDAC with pCR were previously reported during this period.^15^, ^21^ These clinical observations suggest that even pCR does not guarantee “cure” of PDAC and that pCR of PDAC should not signify the end of treatment. Although pCR indicates the maximum grade of response to preoperative treatment and the complete absence of tumor cells in the locoregional area, we should be wary of subclinical distant tumor disseminations in patients with PDAC.^36^, ^37^ In this regard, postoperative adjuvant systemic chemotherapy may have prognostic significance even in pCR cases, although there has been no report addressing this concern. Indeed, in the current study, three of four pCR patients who developed recurrence after surgery did not receive systemic adjuvant chemotherapy, although our results failed to show a significant impact of adjuvant treatment on OS possibly because of the inherent biases regarding delivery of the adjuvant treatment due to the retrospective nature of this study. In contrast, in reports of rectal cancer, giving adjuvant chemotherapy was associated with improved OS in patients with pCR after neoadjuvant treatment.^38^ Further study with a larger patient cohort is required to assess the significance of postoperative adjuvant systemic chemotherapy in pCR cases after preoperative treatment.

The present study has some limitations, including its retrospective nature and the lack of unity in preoperative therapy. Results of this study can be compared with other studies only when patients receive similar perioperative treatments to those carried out in the present study. For further validation of whether our hypothesis concerning pCR is correct in other treatment situations, we must prospectively accumulate pCR data in a multicenter method, referring to whether the cases involve residual cancer cells in the intraductal component.

## 5 CONCLUSION

The minimal residual CIS in cases with the complete absence of an invasive component after preoperative treatment does not yield a prognostic impact, under receiving our perioperative treatment for PDAC. Thus, it would be reasonable to define “pCR” from the prognostic standpoint as follows: the complete absence of an invasive component regardless of the residual CIS. In contrast, minimal residual tumors in the invasive component were significantly associated with unfavorable survival: that is, among the cases with remarkable responses, pCR cases and the cases with even a minimal invasive component should be separately analyzed to more appropriately evaluate the treatment efficacy in the clinical trials of preoperative treatment strategies for PDAC.

## DISCLOSURE

Conflicts of Interest: Authors declare no conflicts of interest for this article.

Funding: We have no financial relationships to disclose.

Author Contributions: Daisaku Yamada made substantial contributions to conception and design, and acquisition of data, and analysis and interpretation of data, drafted the manuscript and gave final approval of the version to be published; Hidenori Takahashi made substantial contributions to conception and design, and acquisition of data, and analysis and interpretation of data, revised the manuscript critically for important intellectual content and gave final approval of the version to be published; Kei Asukai and Osamu Ishikawa made substantial contributions to conception and design, and analysis and interpretation of data, revised the manuscript critically for important intellectual content and gave final approval of the version to be published; Shinichiro Hasegawa and Akira Tomokuni made substantial contributions to conception and design, and acquisition of data, revised the manuscript critically for important intellectual content and gave final approval of the version to be published; Hiroshi Wada, Hirofumi Akita, Masayohi Yasui, and Hiroshi Miyata made substantial contributions to conception and design, and acquisition of data and analysis and interpretation of data, revised the manuscript critically for important intellectual content and gave final approval of the version to be published.
